# Trajectories of acute gastrointestinal injury grade in critically Ill children

**DOI:** 10.1186/s12887-024-04947-0

**Published:** 2024-07-23

**Authors:** Ying Lin, Xiaomin Wang, Kai Zhang, Lijing Wang, Liping Zhang, Junhong Yang

**Affiliations:** 1 Department of Nutrition, Tianjin Children’s Hospital, Tianjin University Children’s Hospital, 225 Longyan Rd, Beichen Dist, Tianjin, China; 2 Pediatric Intensive Care Unit, Tianjin Children’s Hospital, Tianjin University Children’s Hospital, Tianjin, China

**Keywords:** Critically ill children, Gastrointestinal dysfunction, Acute Gastrointestinal Injury grade, Pediatric Intensive Care Unit, Group-based trajectory modeling

## Abstract

**Objective:**

To investigate the characteristics of different Acute Gastrointestinal Injury (AGI) grading trajectories and examine their impact on prognosis in the Pediatric Intensive Care Unit (PICU).

**Methods:**

This retrospective cohort study was conducted at a large children’s hospital in China. The children admitted to the PICU were included. AGI grade was assessed every other day during the initial nine days following PICU admission.

**Results:**

A total of 642 children were included, of which 364 children (56.7%) exhibited varying degrees of gastrointestinal dysfunction (AGI grade ≥ 2). Based on the patterns of AGI grading over time, six groups were identified: low-stable group, low-fluctuating group, medium-decreasing group, medium-increasing group, high-decreasing group, high-persistent group. The high-persistent group accounted for approximately 90% of all recorded deaths. Compared to low-stable group, both the medium-increasing and high-persistent groups exhibited positive correlations with length of stay in PICU (PICU LOS) and length of stay (LOS). Compared to low-stable group, the five groups exhibited a negative correlation with the percentage of energy received by enteral nutrition (EN), as well as the protein received by EN.

**Conclusion:**

This study identified six distinct trajectory groups of AGI grade in critically ill children. The pattern of AGI grade trajectories over time were associated with EN delivery proportions and clinical outcomes.

## Introduction

Gastrointestinal (GI) tract is the most extensive mucosal area within the human body serving not only as a site for digestion but also fulfilling endocrine, immune, and barrier functions. Critically ill children are susceptible to GI tract injury due to factors such as reduced blood flow and inadequate oxygenation. To quantify GI function, the Working Group on Abdominal Problems of the European Society of Intensive Care Medicine has proposed the Acute Gastrointestinal Injury (AGI) grading system, which offers a semiquantitative scoring system ranging from grades 0 to 4 to determine the severity of AGI [1]. The AGI grading system holds significant value in the identification of the extent of GI dysfunction, in both adults and children [2–4]. Previous research studies have primarily concentrated on the worst AGI grade over several days or the assessment of GI function at certain time points. Nevertheless, it is important to note that GI function undergoes dynamic changes throughout the hospitalization period, and previous research fails to adequately consider the correlation between concurrent alterations in GI symptoms and outcomes.

Understanding the progression of GI function during the initial days following admission to the PICU and identifying distinct trajectories of GI dysfunction are crucial. Given the lack of previous research on various trajectories of AGI grading in critically ill children, our study aimed to investigate the characteristics of different AGI grading trajectories and examine their impact on prognosis in the PICU. The results of this investigation will improve physicians’ ability to recognize the probability of unfavorable outcomes and to administer timely treatment.

## Materials and methods

### Study population

This retrospective cohort study was conducted at the Tianjin Children’s Hospital, Tianjin, China, within the period of January 2018 to April 2023. The inclusion criteria for participant selection were as follows: (1) admitted to the PICU with a PICU stay exceeding 48 h; (2) aged 1 to 16 years. The exclusion criteria were as follows: (1) diagnosed with GI diseases; (2) underwent GI surgery; (3) incomplete clinical data.

### Nutrition protocol

If there were no contraindications for enteral nutrition (EN), it was initiated within 24–48 h of admission to the PICU. A team of expert dieticians formulated a nutritional protocol and established target nutrient goals. Resting energy expenditure was estimated using the Schofield equation. The initial infusion rate varied from 10 to 100 mL/h. If enteral nutrition was tolerated without any adverse GI effects, the quantity was be augmented by 20–30 mL/kg/day. The insufficiency of EN in meeting the target energy was supplemented by parenteral nutrition (PN).

### Data collection

Upon admission, demographic data including sex, age, weight, and height were collected. The body mass index for age Z-score (BAZ) was calculated using the WHO Anthro 3.1.0 software. BAZ<-2 was defined as indicating poor nutritional status. On the first day of admission to PICU, the pediatric index of mortality (PIM) 3 score was assessed. The utilization of mechanical ventilation and vasoactive drugs were documented. Blood samples were collected in the morning after admission. Laboratory markers, including serum potassium, total protein, albumin, hemoglobin, C-reactive protein, procalcitonin, interleukin-6, lactate, creatinine, and blood glucose were detected. Hypokalemia was defined as a serum potassium level below 3.5 mmol/L. Throughout hospitalization, a registered dietitian meticulously documented the total energy and protein provided by both EN and PN, as well as the energy and protein specifically derived from EN.

### Assessment of AGI grading

Trained medical staff conducted AGI grading subsequent to admission to the PICU in order to ascertain the precision of the AGI score. All participants in this study underwent AGI grading every other day until their demise, discharge from the hospital, or until the ninth day following admission to the PICU, whichever occurred first. The classification of AGI is shown in Table 1.

**Table 1:** Classification of AGI

Grade	Definition
I (risk of GI dysfunction or failure)	Partial impairment of GI function, manifested as gastrointestinal symptoms related to a known cause and perceived to be transient. Examples: postoperative nausea and/or vomiting during the first few days after abdominal surgery, postoperative absence of bowel sounds, diminished bowel motility in the early phase of shock.
II (GI dysfunction)	The GI tract is unable to perform digestion and absorption adequately to satisfy the nutrient and fluid requirements of the body. There are no changes in the general condition of the patient due to GI problems. Examples: gastroparesis with high gastric residuals or reflux, paralysis of the lower GI tract, diarrhea, intra-abdominal pressure (IAP) 12–15 mmHg, visible blood in gastric content or stool. Feeding intolerance is present if at least 20 kcal/kg BW/day via the enteral route cannot be achieved within 72 h of a feeding attempt.
III (GI failure)	Loss of GI function. Restoration of GI function is not achieved despite interventions, and the general condition is not improving. Examples: persistent feeding intolerance despite treatment manifested as high gastric residuals, persistent GI paralysis, occurrence or worsening of bowel dilatation, IAP, 15–20 mmHg, low abdominal perfusion pressure (below 60 mmHg). Feeding intolerance is present and possibly associated with persistence or worsening of multiple organ dysfunction syndrome.
IV (GI failure with severe impact on distant organ function)	AGI has progressed to become directly and immediately life-threatening, with worsening of multiple organ dysfunction syndrome and shock. Examples: bowel ischemia with necrosis, GI bleeding leading to hemorrhagic shock, Ogilvie syndrome, abdominal compartment syndrome requiring decompression.

### Outcomes

During the period of hospitalization, the rates of all-cause mortality at 28 days and 60 days were documented. Additionally, hospital length of stay (LOS), length of stay in the PICU (PICU LOS), and the occurrence of readmission within 30 days were recorded. The percentage of energy and protein received by EN was calculated by comparing the amount provided by EN to the total energy and protein intake throughout the entire hospitalization period.

### Statistical analysis

Descriptive statistics were reported in the form of frequencies (proportions) for categorical variables, mean ± standard deviation for continuous variables following a normal distribution, and medians (interquartile ranges) for continuous variables that deviated from normal distribution.

Group-based trajectory modeling [5] was employed to identify distinct trajectory groups based on AGI grading for GI injury by GBMT package within the R software. A combination of clinical judgment and the Bayesian Information Criterion (BIC) was employed to determine the number of distinct trajectories. We fitted models with one to eight trajectories. The BIC exhibited a sharp decline as the number of trajectory groups increased up to four. However, when eight groups were considered, the resulting groups fell below the minimum requirement of 5% group size. Beyond six trajectory groups, the decrease in BIC was negligible. The PICU physicians and dietitians determined that six AGI trajectories were more suitable based on their clinical judgment. Ultimately, based on both clinical judgment and BIC, a model with six trajectory groups was selected as the final choice.

In order to evaluate variations in the characteristics of participants and their clinical outcomes linked to each trajectory, the chi-square test, analysis of variance (ANOVA), or Kruskal-Wallis test was employed. All variables were subjected to simple linear regression analysis in order to identify potential predictors of clinical outcomes. Multiple linear regression was conducted to assess the relationship between trajectories and clinical outcomes. To adjust for confounding variables, the multivariate linear model included variables that were statistical associations with clinical outcomes in the simple linear model (*P* < 0.05). Statistical significance was set at a two-sided *P* < 0.05. Statistical analyses were conducted using R software (version 4.1.3, the R Foundation, Vienna, Austria).

## Results

### Characteristics of participants

**Fig. 1:**
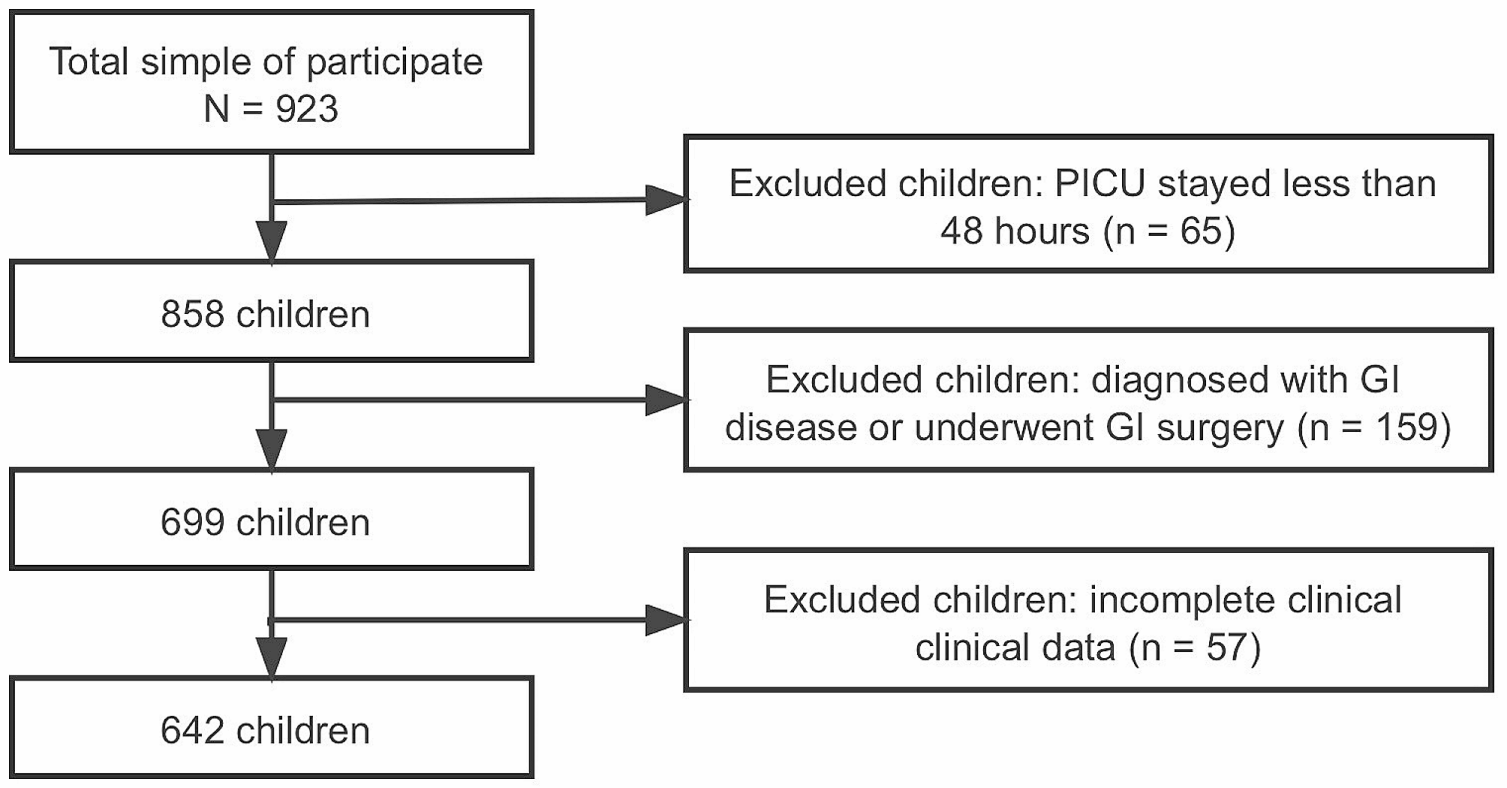
Patient inclusion flowchart

Out of the entire cohort of 923 children aged 1–16 years who were admitted to the PICU and enrolled in the study, a total of 281 children were excluded, including 65 children with a PICU stay of less than 48 h, 159 children diagnosed with GI diseases or who underwent GI surgery, and 57 children with incomplete clinical data. Consequently, the final analysis included a sample size of 642 children, as depicted in Fig. 1.

The final study sample comprised 642 children, of which 274 (42.7%) were male, with a median age of 3 years. Within 9 days of admission to the PICU, 364 children (56.7%) exhibited varying degrees of GI dysfunction (AGI grade ≥ 2). The 28-day in-hospital mortality was 20 (3.1%), and the median PICU LOS was 6 days (interquartile range [4 ~ 7]).

**Fig. 2:**
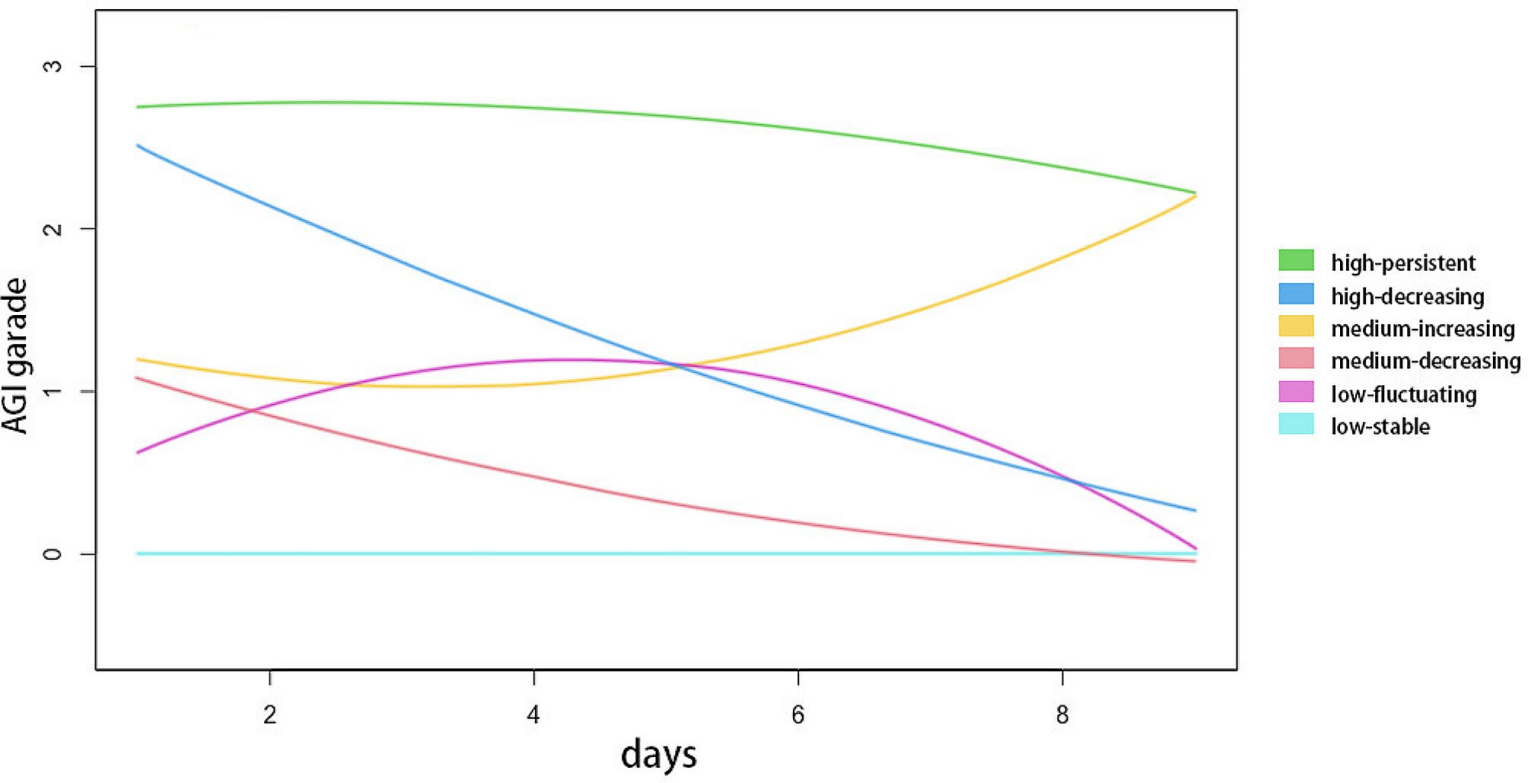
Distinct trajectories of AGI grade over the initial nine days upon PICU admission

The application of group-based trajectory modeling resulted in the identification of six distinct trajectory groups, as depicted in Fig. 2. The characteristics and outcomes of these six groups are presented in Tables 2 and 3. Based on the observed patterns of AGI grading over time, the six groups were labeled: “low-stable” group (*n* = 83, 12.9%), “low-fluctuating” group (*n* = 155, 24.1%),“medium-decreasing” group (*n* = 136, 21.2%), “medium-increasing” group (*n* = 99, 15.4%), “high-decreasing” group (*n* = 102, 15.9%), and “high-persistent” group (*n* = 67, 10.4%).

**Table 2:** Characteristics of distinct trajectory groups in critically ill children

	overall (*n* = 642)	Low-Stable (*n* = 83)	Low-Fluctuating (*n* = 155)	Medium-Decreasing (*n* = 136)	Medium-Increasing (*n* = 99)	High-Decreasing (*n* = 102)	High-Persistent (*n* = 67)	*P*
Demographics								
Age (years)	3 (2,7)	3 (2,5)	3 (2,8)	3 (2,6)	3 (1.75,5.25)	4 (2,10)^acd^	3 (2,7)	0.048
Male, n (%)	274 (42.7)	48 (57.8)	92 (59.4)	74 (54.4)	59 (59.6)	61 (59.8)	34 (50.7)	0.793
BAZ<-2,n (%)	51 (8)	7 (8.4)	13 (8.4)	6 (4.4)	11 (11.1)	9 (8.8)	5 (7.5)	0.566
Principal pathology								
Cardiovascular, n (%)	90 (14)	11 (13.3)	13 (8.4)	15 (11)	21 (21.2)	11 (10.8)	19 (28.4)^bc^	0.001
Neurological, n (%)	127 (19.8)	15 (18.1)	29 (18.7)	21 (15.4)	23 (23.2)	22 (21.6)	17 (25.4)	0.528
Pulmonary, n (%)	250 (38.9)	28(33.7)	52 (33.5)	39 (28.7)	43 (43.4)	53 (52)^bc^	35 (52.2)^c^	0.001
Renal, n (%)	165 (25.7)	21 (25.3)	46 (29.7)	31 (22.8)	23 (23.2)	22 (21.6)	22 (32.8)	0.427
Liver, n (%)	70 (10.9)	13 (15.7)	19 (12.3)	25 (18.4)	3 (3)^ac^	6 (5.9)	4 (6)	0.001
Trauma/burns, n (%)	35 (5.5)	6 (7.2)	9 (5.8)	8 (5.9)	3 (3)	7 (6.9)	2 (3)	0.714
Metabolic disorders, n (%)	113 (17.6)	17 (20.5)	25 (16.1)	27 (19.9)	14 (14.1)	21 (20.6)	9 (13.4)	0.634
Other pathology, n (%)	95 (14.8)	12 (14.5)	28 (18.1)	26 (19.1)	15 (15.2)	9 (8.8)	5 (7.5)	0.106
On the first day of PICU admission								
PIM 3 score	-2.06 (-2.36,-1.76)	-2.06 (-2.36,-2.06)	-2.06 (-2.66,-1.99)	-2.36 (-2.66,-2.06)	-2.06 (-2.36,-1.46)^abc^	-2.06 (-2.36,-1.76)^abc^	-1.76 (-2.06,-0.87)^abcde^	< 0.001
Mechanical ventilation, n (%)	199 (31)	16 (19.3)	39 (25.2)	31 (22.8)	38 (38.4)	43 (42.2)^ac^	32 (47.8)^abc^	< 0.001
Use of vasoactive drug, n (%)	158 (24.6)	9 (10.8)	37 (23.9)	30 (22.1)	28 (28.3)	24 (23.5)	30 (44.8)^abc^	< 0.001
Sepsis, n (%)	101(15.7)	6 (7.2)	22 (14.2)	19 (14)	20 (20.2)	18 (17.6)	16 (23.9)	0.068
Hypokalemia, n (%)	98 (15.3)	5 (6)	16 (10.3)	15 (11)	11 (11.1)	29 (28.4)^abcd^	22 (32.8)^abcd^	< 0.001
Total protein (g/L)	67.35 ± 6.61	67.89 ± 5.59	67.33 ± 6.79	68.27 ± 5.65	66.17 ± 7.48^c^	66.71 ± 7.61	67.62 ± 6	0.19
Albumin (g/L)	42.38 ± 5.26	43.55 ± 3.73	42.44 ± 5.24	43.28 ± 4.06	41.5 ± 6.14a^c^	41.7 ± 6.53^ac^	41.27 ± 5.06^ac^	0.008
Hemoglobin (g/L)	115 (102,124)	116 (101.5,124)	115 (100.75,124)	116 (104.25,126.75)	114.5 (105,125)	113.5 (98.75,122.25)	113 (100,122)	0.308
C-reactive protein (mg/L)	10.6 (2.6,33.1)	13.2 (4.8,40.8)	7.8 (1.9,36.9)	10.4 (3,30.58)	11.25 (2.18,37.8)	11.05 (2.7,31.83)	12.4 (1.5,29.5)	0.536
Procalcitonin (ng/mL)	0.16 (0.08,0.41)	0.18 (0.09,0.38)	0.14 (0.08,0.27)	0.16 (0.09,0.6)	0.15 (0.08,0.4)	0.15 (0.08,0.41)	0.19 (0.07,1)	0.314
Interleukin-6 (pg/mL)	0 (0,2.69)	0 (0,6.52)	0 (0,3.21)	0 (0,0)	0 (0,5.06)	0 (0,0)	0 (0,13.66)	0.083
Serum lactate (mmol/L)	2.49 (2.06,3.37)	2.34 (1.79,3.04)	2.46 (1.97,3.23)	2.34 (1.82,3.07)	2.93 (2.29,3.57)^abc^	2.34 (1.93,3.21)^d^	4.42 (3.21,6.42)^abcde^	< 0.001
Serum creatinine (µmol/L)	27 (22,35)	28 (21.75,33)	27 (22,34.25)	27 (22,35)	27 (20.75,33.25)	26 (21,32.25)	30 (23,46)	0.158
Glucose, n (%) < 6.1mmol/L	342 (53.3)	44 (53)	87 (56.1)	79 (58.1)	51 (51.5)	47 (46.1)	34 (50.7)	0.542
6.1-11.0mmol/L	233 (36.3)	35 (42.2)	50 (32.3)	45 (33.1)	35 (35.4)	43 (42.2)	25 (37.3)	
>11.0mmol/L	67 (10.4)	4 (4.8)	18 (11.6)	12 (8.8)	13 (13.1)	12 (11.8)	8 (11.9)	

^a^ Compared with low-stable group, *P* < 0.05; ^b^ Compared with low-fluctuating group, *P* < 0.05; ^c^ Compared with medium-decreasing group, *P* < 0.05; ^d^ Compared with medium-increasing group, *P* < 0.05; ^e^ Compared with high-decreasing group, *P* < 0.05

BAZ: Body mass index for age Z-score; PIM: Pediatric index of mortality

**Table 3:** Outcomes of distinct trajectory groups in critically ill children

	overall (*n* = 642)	Low-Stable (*n* = 83)	Low-Fluctuating (*n* = 155)	Medium-Decreasing (*n* = 136)	Medium-Increasing (*n* = 99)	High-Decreasing (*n* = 102)	High-Persistent (*n* = 67)	*P*
28-day mortality, n (%)	20 (3.1)	0 (0.0)	0 (0.0)	1 (0.7)	1 (1.0)	0 (0.0)	18 (26.9)^abcde^	< 0.001
60- day mortality, n (%)	24 (3.7)	0 (0.0)	0 (0.0)	1 (0.7)	1 (1.0)	0 (0.0)	22 (32.8)^abcde^	< 0.001
30-day readmission, n (%)	40 (6.2)	3 (3.6)	9 (5.8)	9 (6.6)	9 (9.1)	6 (5.9)	4 (6.0)	0.781
PICU LOS (days)	6 (4,7)	5 (4,6)	6 (4,7)^a^	5 (4,6)^b^	6 (5,12)^abc^	6 (5,8)^acd^	7 (5,14)^abce^	< 0.001
LOS (days)	13 (11,15)	13 (11,15)	13 (11,15)	13 (10,15)	14 (11,19)^bc^	13 (11.75,15)	15 (12,21)^abce^	0.001
Energy received by EN (%)	74 (62,81)	81 (74.75,88)	76 (68,81.25)^a^	78.5 (73,84)^b^	55 (45,65.25)^abc^	76 (70,82)^ad^	19 (14,28)^abcde^	< 0.001
Protein received by EN (%)	72 (61,82)	81.5 (73,100)	73 (67,83)^a^	77.5 (72,100)^b^	55 (42,63)^abc^	77 (70,82)^ad^	16 (10,28)^abcde^	< 0.001

^a^ Compared with low-stable group, *P* < 0.05; ^b^ Compared with low-fluctuating group, *P* < 0.05; ^c^ Compared with medium-decreasing group, *P* < 0.05; ^d^ Compared with medium-increasing group, *P* < 0.05; ^e^ Compared with high-decreasing group, *P* < 0.05

PICU LOS: Length of stay in PICU; LOS: Length of stay

#### Group 1: low-stable

This group with a stable low AGI grading was characterized by lower levels of PIM3 score, serum albumin and lactate, as well as a lower incidence of mechanical ventilation, utilization of vasoactive drug, and hypokalemia upon admission to the PICU. The outcomes for this group were overall favorable, with a lower PICU LOS and 0% in-hospital mortality. Additionally, this cohort received the highest percentage of energy/protein through EN.

#### Group 2: low-fluctuating

The children within this group initially displayed low AGI grading, which subsequently fluctuated before ultimately returning to low levels. The characteristics and mortality rates were comparable to those of the low-stable group. However, it is worth noting that this group exhibited an extended PICU LOS and a lower rate of energy/protein intake through EN when compared to the low-stable group.

#### Group 3: medium-decreasing

This group initially exhibited moderate levels of AGI grading and experienced a reduction in GI symptoms. Notably, this group displayed the greatest resemblance to the low-stable group in terms of characteristics and outcomes. Furthermore, the outcomes were even more advantageous compared to the low-fluctuating group, as evidenced by a shorter PICU LOS and a higher rate of energy/protein intake through EN.

#### Group 4: medium-increasing

The initial AGI grading of these children was moderate and showed a progressive increase. This group was characterized by higher PIM3 scores, lower levels of total protein, albumin, and lactate in comparison to the low-stable group. The outcomes were notably worse than those observed in the aforementioned groups.

#### Group 5: high-decreasing

The initial AGI grading levels of this group upon admission to the PICU were high, but decreased over time. In comparison to the medium-increasing group, these children had shorter PICU LOS and received a higher rate of energy/protein through EN.

#### Group 6: high-persistent

The group exhibited the most elevated levels of AGI grading, which remained consistently high throughout hospitalization. Notably, these children displayed the highest PIM3 score and lactate levels, as well as the highest rates of mortality at both the 28-day and 60-day marks. Additionally, this group demonstrated the lowest proportion of energy/protein intake provided by EN.

### The association of different trajectories with outcomes

A univariate analysis was conducted on the outcome characteristics, as presented in Table 4.

**Table 4:** Univariate analysis on the outcomes of critically ill children by simple linear regression

	Length of PICU stay			Length of stay			Energy received by EN			Protein received by EN	
	*β* (*95%CI*)	*P*		*β* (*95%CI*)	*P*		*β* (*95%CI*)	*P*		*β* (*95%CI*)	*P*
Age	-0.001 (-0.113,0.111)	0.985		0.064 (-0.069,0.197)	0.348		0.074 (-0.342,0.489)	0.728		0.058 (-0.418,0.535)	0.810
Male	0.421 (-0.449,1.290)	0.342		0.485 (-0.546,1.516)	0.356		-0.326 (-3.543,2.892)	0.842		0.937 (-2.749,4.624)	0.618
BAZ<-2	1.98 (0.395,3.565)	0.014		1.522 (-0.363,3.406)	0.113		-2.636 (-8.517,3.246)	0.379		-2.238 (-8.976,4.501)	0.515
Cardiovascular	-0.729 (-1.967,0.509)	0.248		-0.494 (-1.964,0.975)	0.509		-10.884 (-15.389,-6.379)	< 0.001		-11.865 (-17.037,-6.693)	< 0.001
Neurological	0.696 (-0.383,1.775)	0.206		0.576 (-0.704,1.856)	0.377		-1.525 (-5.518,2.468)	0.453		-2.667 (-7.241,1.907)	0.253
Pulmonary	0.849 (-0.031,1.729)	0.059		1.877 (0.841,2.913)	< 0.001		-5.734 (-8.967,-2.501)	0.001		-7.567 (-11.261,-3.873)	< 0.001
Renal	0.794 (-0.189,1.776)	0.113		1.502 (0.341,2.664)	0.011		-0.892 (-4.533,2.748)	0.630		-0.728 (-4.902,3.445)	0.732
Liver	-1.052 (-2.431,0.326)	0.134		-1.507 (-3.14,0.126)	0.070		6.929 (1.852,12.006)	0.008		8.068 (2.25,13.886)	0.007
Trauma/burns	-0.629 (-2.524,1.265)	0.515		-1.813 (-4.056,0.43)	0.113		0.864 (-6.145,7.872)	0.809		2.436 (-5.595,10.467)	0.552
Metabolic disorders	-0.161 (-1.29,0.969)	0.780		0.354 (-0.986,1.693)	0.604		3.843 (-0.325,8.01)	0.071		5.577 (0.807,10.346)	0.022
Other pathology	-0.137 (-1.349,1.075)	0.825		-0.517 (-1.953,0.92)	0.480		4.082 (-0.388,8.553)	0.073		4.083 (-1.044,9.21)	0.118
PIM 3 score	1.173 (0.522,1.824)	< 0.001		1.006 (0.23,1.782)	0.011		-11.414 (-13.678,-9.15)	< 0.001		-12.24 (-14.86,-9.621)	< 0.001
Mechanical ventilation	1.168 (0.242,2.094)	0.013		2.576 (1.491,3.661)	< 0.001		-7.013 (-10.41,-3.615)	< 0.001		-9.21 (-13.088,-5.331)	< 0.001
Use of vasoactive drug	1.577 (0.586,2.568)	0.002		0.989 (-0.193,2.171)	0.101		-7.021 (-10.675,-3.367)	< 0.001		-7.311 (-11.507,-3.115)	0.001
Sepsis	0.92 (-0.26,2.099)	0.126		-0.046 (-1.447,1.355)	0.949		-4.697 (-9.052,-0.342)	0.035		-4.957 (-9.952,0.037)	0.052
Hypokalemia	-0.119 (-1.315,1.077)	0.845		0.236 (-1.182,1.655)	0.744		-6.734 (-11.127,-2.34)	0.003		-7.945 (-12.978,-2.911)	0.002
Total protein	0.014 (-0.051,0.080)	0.666		0.052 (-0.025,0.129)	0.186		0.252 (0.012,0.492)	0.040		0.304 (0.029,0.579)	0.030
Albumin	0.044 (-0.038,0.125)	0.296		0.056 (-0.041,0.153)	0.254		0.567 (0.267,0.867)	< 0.001		0.629 (0.285,0.973)	< 0.001
Hemoglobin	-0.029 (-0.055,-0.004)	0.024		-0.030 (-0.060,0.000)	0.054		0.055 (-0.039,0.15)	0.249		0.056 (-0.052,0.164)	0.312
C-reactive protein	0.004 (-0.008,0.016)	0.475		0.006 (-0.008,0.021)	0.376		-0.007 (-0.052,0.037)	0.745		-0.025 (-0.076,0.026)	0.337
Procalcitonin	-0.038 (-0.099,0.023)	0.228		-0.019 (-0.091,0.054)	0.615		-0.146 (-0.372,0.079)	0.203		-0.175 (-0.434,0.083)	0.183
Interleukin-6	0 (-0.002,0.001)	0.628		-0.001 (-0.004,0.001)	0.254		-0.017 (-0.024,-0.01)	< 0.001		-0.017 (-0.025,-0.009)	< 0.001
Lactic acid	0.553 (0.235,0.872)	0.001		0.555 (0.176,0.933)	0.004		-7.834 (-8.855,-6.813)	< 0.001		-8.662 (-9.847,-7.478)	< 0.001
Creatinine	0.022 (0.004,0.041)	0.018		0.035 (0.013,0.057)	0.002		-0.141 (-0.209,-0.073)	< 0.001		-0.165 (-0.243,-0.087)	< 0.001
Glucose	0.515 (-0.123,1.152)	0.113		0.458 (-0.299,1.215)	0.235		-0.546 (-2.908,1.816)	0.650		-0.162 (-2.87,2.546)	0.907
Low-Stable	reference			reference			reference			reference	
Low-Fluctuating	1.223 (-0.146,2.593)	0.080		0.017 (-1.675,1.709)	0.984		-5.983 (-8.985,-2.981)	< 0.001		-8.121 (-11.873,-4.368)	< 0.001
Medium-Decreasing	-0.123 (-1.525,1.28)	0.864		-0.886 (-2.619,0.847)	0.316		-2.857 (-5.931,0.218)	0.069		-3.666 (-7.509,0.177)	0.061
Medium-Increasing	3.845 (2.347,5.344)	< 0.001		2.348 (0.497,4.200)	0.013		-26.51 (-29.796,-23.225)	< 0.001		-31.27 (-35.376,-27.164)	< 0.001
High-Decreasing	1.326 (-0.163,2.815)	0.081		-0.028 (-1.867,1.811)	0.976		-5.224 (-8.487,-1.961)	0.002		-8.051 (-12.129,-3.972)	< 0.001
High-Persistent	6.9 (5.246,8.554)	< 0.001		5.336 (3.293,7.379)	< 0.001		-56.977 (-60.602,-53.352)	< 0.001		-63.004 (-67.535,-58.473)	< 0.001

BAZ: Body mass index for age Z-score; PIM: Pediatric index of mortality; PICU LOS: Length of stay in PICU; LOS: Length of stay

After adjusting for these confounding factors (*P* < 0.05 in simple linear regression analysis), the results of the multivariate linear regression model were presented in Table 5. Compared to low-stable group, both the medium-increasing and high-persistent groups exhibited positive correlations with PICU LOS and LOS; whereas null significant associations were observed for other three groups. Compared to low-stable group, the five groups exhibited a negative correlation with the percentage of energy received by EN, as well as the protein received by EN.

**Table 5:** Association between trajectory groups and outcomes in critically ill children

	PICU LOS			LOS			Energy received by EN			Protein received by EN	
	*β* (*95%CI*)	*P*		*β* (*95%CI*)	*P*		*β* (*95%CI*)	*P*		*β* (*95%CI*)	*P*
Low-Stable	reference			reference			reference			reference	
Low-Fluctuating	1.199 (-0.174,2.573)	0.087		-0.181 (-1.862,1.501)	0.833		-5.725 (-8.677,-2.774)	< 0.001		-5.652 (-8.601,-2.704)	< 0.001
Medium-Decreasing	-0.09 (-1.497,1.316)	0.900		-1.008 (-2.729,0.712)	0.250		-3.263 (-6.281,-0.244)	0.034		-3.227 (-6.240,-0.213)	0.036
Medium-Increasing	3.897 (2.367,5.427)	< 0.001		2.056 (0.182,3.930)	0.032		-24.639 (-27.960,-21.317)	< 0.001		-24.529 (-27.841,-21.217)	< 0.001
High-Decreasing	1.218 (-0.288,2.723)	0.113		-0.452 (-2.294,1.389)	0.630		-4.419 (-7.706,-1.132)	0.008		-4.368 (-7.646,-1.090)	0.009
High-Persistent	7.401 (5.491,9.311)	< 0.001		5.044 (2.706,7.383)	< 0.001		-50.915 (-55.114,-46.716)	< 0.001		-50.815 (-54.996,-46.633)	< 0.001

PICU LOS: Length of stay in PICU; LOS: Length of stay

## Discussion

Owing to the critical condition and unstable hemodynamics of patients in the PICU, GI damage is often observed to varying degrees. Studies have indicated that GI dysfunction not only constitutes a component of Multiple Organ Dysfunction Syndrome, but also serves as its initiator [6]. In India, 47% of critically ill children developed AGI within the first week of admission [3]. In China, the incidence of children with severe community-acquired pneumonia happened AGI at 71.9% [7]. In this study, the occurrence of AGI within 9 days after PICU admission was 56.7%, indicating a high prevalence of AGI in the PICU that warrants attention. This study uncovered distinct clinical characteristics and divergent outcomes among critically ill children based on different trajectories of AGI grading.

The findings of our study revealed notable variations in age distribution, principal pathology types, PIM3 score, mechanical ventilation, vasoactive drug usage, hypokalemia, albumin levels, and lactate levels among the different groups. Specifically, the high-persistent group exhibited a higher PIM3 score, lower albumin levels, higher serum lactate levels, a greater likelihood of mechanical ventilation usage, vasoactive drug usage, and hypokalemia compared to the other groups. The PIM3 score, derived from the PIM2 score in 2013, demonstrates enhanced prognostic accuracy for mortality risk among pediatric patients receiving intensive care [8]. The relationship between critical illness scores and GI function has been demonstrated in adult studies [9, 10]. A higher PIM3 score indicates a more severe condition and exhibits a correlation with GI dysfunction in PICU children. A lower serum albumin level is thought to be indicative of malnutrition. The previous study has proved malnutrition can augment intestinal permeability through the alteration of the mucosal immune barrier [11]. Reduced levels of serum albumin are linked to intestinal wall edema, which will lead to bowel dysfunction [12]. A study revealed that an elevation in continuous positive airway pressure is correlated with a reduction in microvascular oxygen saturation within the gastric mucosa, potentially impairing the functioning of the GI tract [13]. The depletion of potassium impedes the activity of Na-K-ATPase, thereby causing heightened permeability of epithelial cells [14]. Recent findings indicated a potassium-deficient diet can result in increased intestinal permeability [15]. Serum lactate level serves as a reliable indicator of impaired tissue perfusion. Elevated serum lactate levels indicate the presence of hypoxia in tissues and GI dysfunction in patients with hemodynamic instability [16]. Administration of vasoactive medications can induce a reduction in blood supply to the digestive tract, resulting in delayed gastric emptying and diminished intestinal peristalsis [17, 18].

Numerous investigations have substantiated that a higher AGI grade was an independent risk factor for mortality in both adult and pediatric populations [2, 3, 19–21], as well as the PICU stay and mechanical ventilation [2, 22–25]. However, these investigations failed to consider the duration and progression of AGI. In our study, we observed differences in outcomes based on different AGI grade trends. Specifically, the high-persistent group had the highest mortality at 28-day and 60-day intervals, while the high-decreasing group did not experience any deaths. Compared to the low-stable group, both the medium-increasing and high-persistent groups had longer stays in PICU and overall hospitalization. It is worth noting that the medium-increasing group experienced an extended PICU LOS and LOS, in contrast to the medium-decreasing and high-decreasing groups. Initially poor GI function may represent a reversible state of physiological stress or insult. When addressed and managed effectively, it can lead to recovery and improved outcomes. Conversely, worsening GI function may indicate a more severe or advanced state of illness that is less responsive to treatment, thereby potential associated with critical illness. This interpretation suggests that the AGI trajectory, particularly the ability to improve over time, is a critical factor in patient outcomes and highlights the necessity for close monitoring and proactive management of GI health in critically ill patients.

The findings of our study suggest a correlation between AGI trajectories and EN intake. Compared to the low-stable group, the other groups, such as the low-fluctuating group, exhibited a negative correlation with the percentage of energy/protein received by EN. The low-stable group consistently received a more adequate and stable provision of energy and protein via EN, suggesting effective nutritional support. In contrast, other groups, including the low-fluctuating group, might have experienced variations in EN administration, possibly due to intermittent feeding challenges or metabolic changes, leading to a lower percentage of energy/protein intake relative to total needs. The intestine is the largest immune organ in humans, which regulates various functions [26]. EN plays a crucial role in enhancing patients’ immune function [27, 28]. According to a previous study, EN interruption is linked to an extended PICU LOS and a lower success rate in achieving energy target [29]. This correlation was further supported by our study, where the group with a lower proportion of EN delivery experienced a prolonged PICU LOS and overall LOS.

The present study has some limitations. Firstly, our findings are based on a single-center experience, which implies that other centers may encounter distinct issues specific to their respective institutions. Secondly, the sample size was insufficient due to the study being conducted at a single center. Thirdly, the duration of observation for AGI did not seem to be extensive enough to comprehend long-term patterns in GI symptoms. Consequently, it is imperative to conduct multicenter studies with large sample sizes and prolonged observation periods in order to validate these results. Fourthly, In order to mitigate potential bias, we conducted additional multivariate analyses that accounted for various factors, such as disease status, PIM3 score, use of mechanical ventilation and vasoactive drugs, presence of hypokalemia, levels of total protein, albumin, interleukin-6, lactate, creatinine. Nevertheless, the presence of unmeasured confounders may still impact the results of our study. For instance, the relationship between patient disease dynamics, AGI score, and EN intake warrants further investigation in subsequent research.

Our study underscores the importance of regular monitoring of GI function through a combination of clinical assessments, laboratory markers such as serum albumin and lactate levels, and imaging when indicated, rather than solely relying on AGI grade at PICU admission. By observing the changing trend of GI symptoms, as opposed to a certain time point, a more accurate clinical prognosis can be obtained. Early identification of AGI trajectory can prompt timely interventions, potentially preventing progression to severe GI complications. Early intervention, including tailored nutritional support, fluid and electrolyte management, medications such as proton pump inhibitors and motility agents, and pain management, is crucial to improve outcomes in critically ill children.

## Conclusion

This study identified six distinct trajectory groups of AGI grade during the initial nine days following admission to the PICU in critically ill children. The medium-increasing and high-persistent groups demonstrated a positive correlation with the LOS both in the PICU and overall hospitalization. It is important to note that these findings underscore the complexity of AGI in pediatric patients and suggest that monitoring the progression of AGI grades is essential for predicting and managing clinical outcomes effectively. These findings affirm the importance of continuously monitoring GI symptoms in critically ill children, rather than relying solely on a certain time point of PICU admission.

## Data Availability

The datasets used during the current study are available from the corresponding author on reasonable request.
